# Phenotype heterogeneity of congenital adrenal hyperplasia due to genetic mosaicism and concomitant nephrogenic diabetes insipidus in a sibling

**DOI:** 10.1186/s12881-018-0629-2

**Published:** 2018-07-11

**Authors:** Yılmaz Kor, Minjing Zou, Roua A. Al-Rijjal, Dorota Monies, Brian F. Meyer, Yufei Shi

**Affiliations:** 1 Pediatric Endocrinology Division, Ministry of Health, Adana Public Hospitals Association, Adana City Hospital, Adana, Turkey; 2 Department of Genetics (MBC-03), King Faisal Specialist Hospital and Research Centre, P.O. Box 3354, Riyadh, 11211 Saudi Arabia

**Keywords:** 21-hydroxylase deficiency, Mosaicism, Nephrogenic diabetes insipidus, CYP21A2, AQP2

## Abstract

**Background:**

Congenital adrenal hyperplasia (CAH) due to 21-hydroxylase deficiency (21OHD) is an autosomal recessive disorder caused by mutations in the *CYP21A2*. Congenital nephrogenic diabetes insipidus (NDI) is a rare X-linked recessive or autosomal recessive disorder caused by mutations in either *AVPR2* or *AQP2*. Genotype-phenotype discordance caused by genetic mosaicism in CAH patients has not been reported, nor the concomitant CAH and NDI.

**Case presentation:**

We investigated a patient with concomitant CAH and NDI from a consanguineous family. She (S-1) presented with clitoromegaly at 3 month of age, and polydipsia and polyuria at 13 month of age. Her parents and two elder sisters (S-2 and S-3) were clinically normal, but elevated levels of serum 17-hydroxyprogesterone (17-OHP) were observed in the mother and S-2. The coding region of *CYP21A2* and *AQP2* were analyzed by PCR-sequencing analysis to identify genetic defects. Two homozygous *CYP21A2* mutations (p.R357W and p.P454S) were identified in the proband and her mother and S-2. The apparent genotype-phenotype discordance was due to presence of small amount of wild-type *CYP21A2* alleles in S-1, S-2, and their mother’s genome, thus protecting them from development of classic form of 21OHD (C21OHD). A homozygous *AQP2* mutation (p.A147T) was also found in the patient. The patient was treated with hydrocortisone and hydrochlorothiazide. Her symptoms were improved with normal laboratory findings. The clitoromegaly is persisted.

**Conclusions:**

Genetic mosaicism is a novel mechanism contributing to the genotype-phenotype discordance in 21OHD and small percentage of wild-type *CYP21A2* alleles may be sufficient to prevent phenotype development. This is a first report of concurrent 21OHD and NDI caused by simultaneous homozygous *CYP21A2* and *AQP2* mutations.

## Background

Congenital adrenal hyperplasia (CAH) is a group of autosomal recessive disorders of steroid metabolism caused by the deficiency (loss of or severely decreased activity) of one of five steroidogenic enzymes involved in the cortisol biosynthesis: cholesterol side-chain cleavage enzyme (CYP11A1), 3 beta-hydroxysteroid dehydrogenase (HSD3B2), 17 alpha-hydroxylase/17,20 lyase (CYP17A1), 21-hydroxylase (CYP21A2), and 11 beta-hydroxylase (CYP11B1) [1]. More than 95% of CAH cases are from steroid 21-hydroxylase deficiency (21OHD, MIN 201910) due to mutations in the *CYP21A2* gene [2–4].

Steroid 21-hydroxylase catalyzes the conversion of 17-hydroxyprogesterone (17-OHP) to 11-deoxycortisol, and progesterone to 11-deoxycorticosterone. Its deficiency leads to overproduction and accumulation of 17-OHP. This results in excessive androgen production and decreased aldosterone and cortisol synthesis. Based on the clinical severity, CAH can be classified into classic form of 21OHD (C21OHD) and the mild non-classic form of 21OHD (NC21OHD). C21OHD is estimated to occur with a frequency of 1 in 7000 to 15,000 live births in most populations and can be further divided into salt-wasting (70%) and simple virilizing form (30%) [3, 5]. The salt-wasting form is characterized by severe renal salt loss as a consequence of aldosterone deficiency whereas the simple virilizing form is characterized by virilization of external genitalia (clitoromegaly in females and penile growth in males) and precocious pseudopuberty as a result of adrenal androgen overproduction. NC21OHD occurs approximately in a frequency of 1 in 1000 live births in various Caucasian populations and is predominantly in female patients with pseudopuberty, acne, hirsutism, and decreased fertility [5]. It is often discovered during studies of family members who have classic form of the disease. In contrast to C21OHD, it is generally believed that patients with NC21OHD have mild mutations on both alleles or one severe and one mild mutations of *CYP21A2* that retain some of 21-hydroxylase activity. For example, missense mutations in exon 1 (P30L), exon 7 (V281 L), exon 8 (R339H), or exon 10 (P454S) are found in NC21OHD patients with enzymatic activity of about 20–60% of wild-type [6]. Although there is a strong genotype-phenotype correlation in 80–90% of cases [4, 7], discordance can still be found in remaining patients [8, 9].

Diabetes insipidus is defined as the passage of large volumes (> 3 L/24 h or 2 L/m^2^/24 h) of dilute urine with reduced urine osmolality (< 300 mOsm/kg) and is caused by reduced or absent secretion of pituitary antidiuretic hormone arginine vasopressin or by a poor kidney response to the hormone. Polyuria with hyposthenuria and polydipsia are the main hallmarks of the disease. Congenital nephrogenic diabetes insipidus (NDI) is a rare genetic disorder of kidney characterized by a defect to concentrate urine despite normal or elevated arginine vasopressin [10]. About 90% of patients are males with X-linked recessive NDI (OMIM 304800) caused by mutations in the arginine-vasopressin receptor 2 gene (*AVPR2*). In 10% of patients, the disease is caused by mutations in the aquaporin-2 gene (*AQP2*) and transmitted as an autosomal-recessive or autosomal-dominant (OMIM 125800) mode [11–13].

In the present study, we characterized a family with concomitant NC21OHD and NDI, and identified genetic mosaicism as a new mechanism causing phenotype heterogeneity of 21OHD.

## Case presentation

The proband (S-1 in Table 1) was first admitted to the hospital due to clitoromegaly at 3 months of age. She was born with normal vaginal delivery at 39 weeks of pregnancy, and weighed 3500 g. She was a third child of consanguineous parents who are first-degree cousins. Her mother had a history of menstrual irregularity and mild hirsutism, and was diagnosed polycystic ovary syndrome at a different hospital. Her two elder sisters were normal with regular menstrual cycle and breast development Tanner stage IV, axillary and pubic hair Tanner stage V. Physical examination showed clitoromegaly (1.5 cm) without urogenital sinus and hyperpigmentation. Laboratory tests showed elevated 17-OHP (6.3 ng/ml, normal 0.2–0.9 ng/ml), normal ACTH (10 pg/ml, normal: 10–60 pg/ml) and cortisol 9.6 μg/dl (normal: 6.2–19.4 μg/dl), reduced aldosterone (< 3.7 ng/dl, normal: 3.7–43.7 ng/dl). 17-OHP was > 19.2 ng/ml after a standard dose of adrenocorticotropic hormone stimulation test (ACTH, 250 μg/m^2^). She was diagnosed as NC21OHD due to mild phenotype (see discussion and conclusions) and treated with hydrocortisone 15 mg/m^2^/day. At the age of 13 months, she was admitted to the hospital again due to polyuria and polydipsia. Urine density was 1.002 (normal: 1.005–1.030). Hydrochlorothiazide was started at 2 mg/kg/d when she was not responded to desmopressin acetate treatment. At the same time, fludrocortisone (100 mcg/m^2^/day) was added due to mild hyponatremia (133 mmol/L, normal: 136–145 mmol/L) and hyperpotassemia (5.6 mmol/L, normal 3.5–5.1). Fludrocortisone treatment was discontinued after genetic analysis confirmed the diagnosis. Under the hydrocortisone treatment, the laboratory findings were stable. However, clitoromegaly is persisted and clitoroplasty is planned when she is 9–10 years old.

**Table 1:** Clinical and genetic analysis of a patient with concomitant 21-hydroxylase deficiency and nephrogenic diabetes insipidus

	Sex	Age (year)	Clinical presentations	Biochemical tests						AQP2	CYP21A2	
				Renin (μIU/ml)	Aldosterone (ng/dl)	17-OHP (ng/ml)	ACTH (pg/ml)	Cortisol (μg/dl)	24-h urine osmolality	c.439G >A, p.A147T in Exon2	c.1069C >T,p.R357W in Exon8	c.1360C >T, p.P454S in Exon10
Father	M	44	normal	64	8	0.56	51.25	12.1	855	het	het	het
Mother	F	40	PCOS with menstrual irregularity and mild hirsutism	86	< 3.7	2.01	25.73	8.8	737	het	homo	homo
S-1	F	7	Clitoromegaly, polyuria, polydipsia	115	< 3.7	6.3	10	9.6	87	homo	homo	homo
				621^a^	54^a^	0.51^a^	5.15^a^	22.6^a^				
S-2	F	11	normal	47	< 3.7	1.87	34.65	9.3	473	wt	homo	homo
S-3	F	13	normal	161	20	0.51	13.5	7.4	800	wt	wt	wt
Normal range				5.3–99	3.7–43.7	0.2–0.9	10–60	6.2–19.4	500–800 mOsm/kg of water			

^a^under hydrocortisone treatment at 15 mg/m^2^/day

**Fig. 1:**
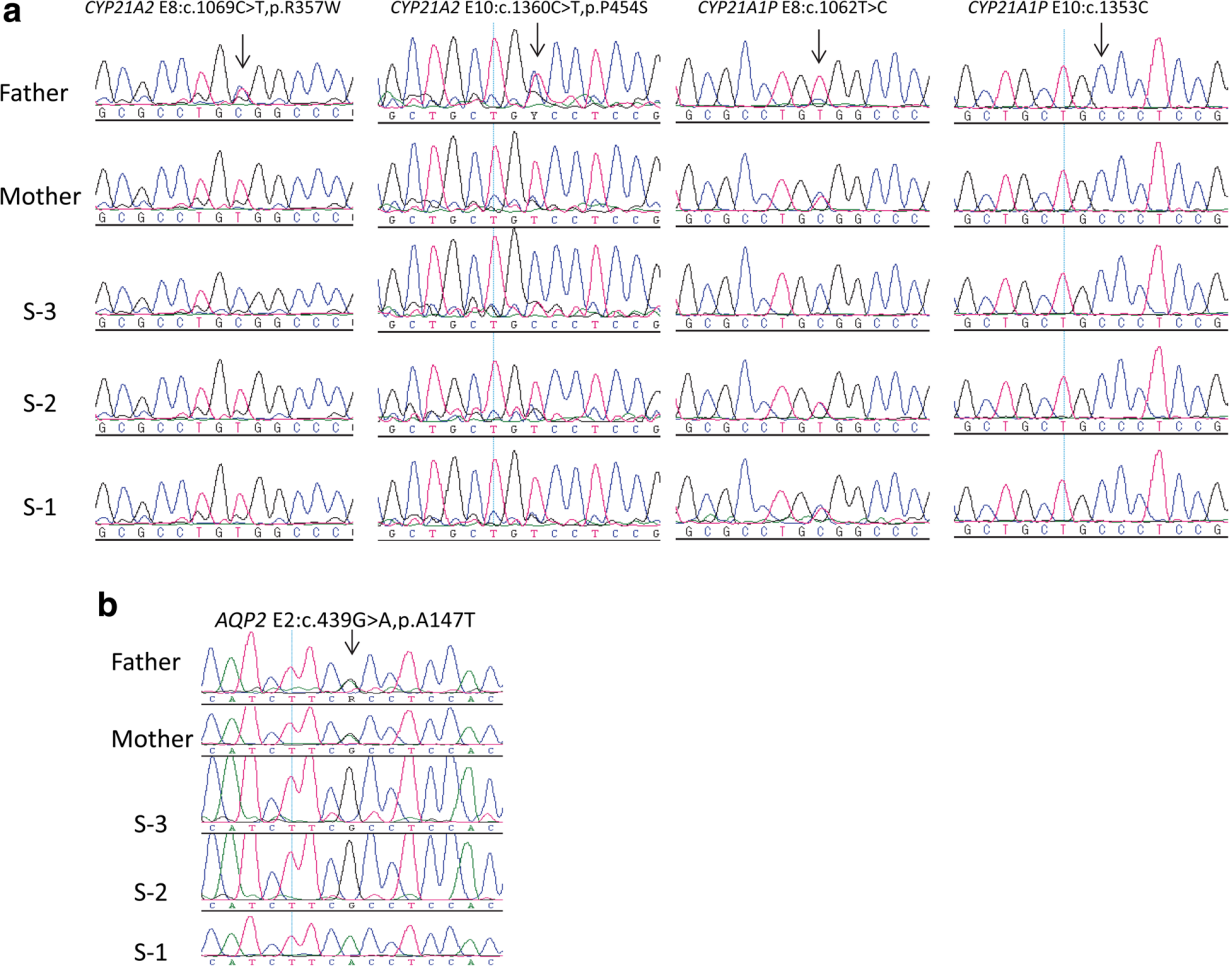
Sequence analysis of *CYP21A2, CYP21A1P* and *AQP2* in patient and family members. **a** Homozygous *CYP21A2* c.1069C >T (p.R357W) in exon 8 and c.1360C>T (p.P454S) in exon 10 are present in patient (S-1), her unaffected mother and sister S-2. Father is a heterozygous carrier and sister S-3 is normal. Mutations and corresponding *CYP21A1P* pseudogene sequences are indicated by an arrow. A pseudogene-specific primer pair was used to amplify 2.5-kb *CYP21A1P* gene containing exons 3–10 (F: 5’-CGGACCTGTCGTTGGTCTCTG-3′, R: 5’-GATTAAGCCTCAATCCTCTGCGGCA-3′). The resulting PCR products were re-amplified using the common *CYP21A2* primer pairs for exon 8 (F: 5’-TTGCTGAGGGAGCGGCTGGAG-3′, R: 5’-GTTAGAGGCTGGCCAGGACCT-3′) and exon 10 (F: 5-TGAAAATGTGGTGGAGGCTGG-3′, R: 5’-CTCGCAGCACTGTGTTTACA-3′) followed by direct sequencing of PCR products. **b** A homozygous *AQP2* c.439G>A (p.A147T) in exon 2 is shown in patient. Her parents are heterozygous carriers

Molecular characterization of *CYP21A2* mutation. Genomic DNA from peripheral blood leukocytes of patient and family members was isolated using Gentra Blood Kit (Qiagen Corp, CA). To obtain *CYP21A2* gene (NM_000500) free from its pseudogene (*CYP21A1P*) which shares 98% sequence homology, a differential primer pair was used to amplify the entire 3.5-kb gene containing 10 exons of *CYP21A2* (F: 5’-CCCAGGTGGGGGCGGACACTA-3′, R: 5’-AATTAAGCCTCAATCCTCTGCAGCG-3′) [14]. The long-range PCR was performed using Extensor Hi-Fidelity PCR Master Mix (cat #AB-0794/B, Thermo Scientific, Waltham, MA, USA). The PCR conditions were 94 °C for 2 min followed by 30 cycles of amplification (94 °C for 10 s, 60 °C for 30 s, 68 °C for 2 min) with final extension at 68 °C for 7 min. The PCR products were re-amplified using the universal primer pairs for each of 10 exons [14]. The resulting PCR products were directly sequenced using an automated ABI PRISM 3700 sequencer (Foster City, CA, USA) or cloned into a TA vector (Invitrogen, CA, USA). Individual clones were subsequently sequenced. Two previously reported homozygous mutations in the *CYP21A2* were identified in the patient: c.1069C >T, p.R357W in Exon 8 and c.1360C >T, p.P454S in Exon10 (Fig. 1a), confirming that the patient had 21OHD. Unexpectedly, the same homozygous mutations were also present in her mother and elder sister S-2 who had no clinical symptoms except for menstrual irregularity and mild hirsutism in the mother and precocious puberty in S-2. Lab tests showed low aldosterone and mild increase of 17-OHD (Table 1), but their Na and K levels were normal. Father was a heterozygous carrier and elder sister S3 was wild-type. To further substantiate the lab tests, ACTH stimulation test was conducted again in 2017 including all family members. As shown in Table 2, the post stimulation 17-OHP levels were about 10-fold higher in the mother, 7-fold higher in S-2, and 20-fold higher in patient S-1 when compared to the father or S-3. These data suggest that the mother and S-2 had mild NC21OHD as well. The apparent wild-type genotype in S-3 could not be explained given that the mother was homozygous and father heterozygous for the p.R357W and p.P454S mutants. Genetic mosaicism may contribute to the genotype discordance and phenotype variations among family members: the mother may carry a wild-type *CYP21A2* allele which was passed on to her children. Another possibility was that the mother may not be real biological mother of S3. To confirm whether the parents and S-3 were related, we conducted forensic analysis on the family members by multiplex STR analysis using AmpFlSTR®Identifiler® PCR amplification kit (ABI, Foster City, CA, USA). Capillary electrophoresis and data analysis were carried out by Genetic Analyzer 3130 (ABI, Foster City, CA, USA) and GeneMapper ID software version 3.2 [15]. As shown in Table 3, there was no maternal or paternal exclusion for S-1 and S-3 among 16 STR locus, indicating that both S-1 and S-3 were biological daughters of the parents. To explore the possibility of genetic mosaicism at the *CYP21A2* locus, we cloned PCR products of exons 8 and 10 from the mother, S-1 and her elder sister S-2, and sequenced more than 55 individual clones to determine if we were able to find small number of wild-type clones that could not be detected by direct PCR-sequencing analysis. Indeed, 5 wild-type clones were found among 94 clones sequenced for R357W (5.3%, 5/94), and 8 wild-type clones was found among 117 clones sequenced for P454S (6.8%, 8/117) in the mother (Table 4). Although no wild-type clone for P454S was detected in both S-1 and S-2, wild-type clones for R357W were found in S-1 (3/118, 2.5%) and S-2 (2/58, 3.4%). These data suggest that wild-type alleles in the mother’s genome (about 6% in average) may be passed on to S-3, resulting in wild-type genotype. The phenotype variations in CAH among the mother, S-1 and S-2 likely depend on the abundance of wild-type alleles present in each individual.

**Table 2:** ACTH stimulation test among patient and family members

	Father			Mother			S-1			S-2			S-3		
	0’	30’	60’	0’	30’	60’	0’	30’	60’	0’	30’	60’	0’	30’	60’
Glucose	114			98			82			93			87		
Na	142			140			142			141			142		
K	4.2			4.6			3.9			5			4		
ACTH	35			13.7			1.88			9.3			30		
Cortisol (μg/dL)	10.8	24.8	28.3	6.7	9.8	18	5.6	4.7	8	9.2	16.2	18.3	9.4	32.2	33.3
17-OHP (ng/ml)	0.9	1.8	1.2	0.8	16.6	10.4	1.1	28	33	2.1	9.7	10.8	0.9	1.2	1.62

**Table 3:** Forensic analysis of family members using 16 STR markers

STR	Father		Mother		S-1		S-3	
D8S1179	13		12	14	12	13	12	13
D21S11	28	29.2	28.2	33.2	28	33.2	29.2	33.2
D7S820	8.2	9.2	11		9.2	11	9.2	11
CSFIP0	12	13	10.2	12	10.2	13	12	
D3S1358	13	15.2	14		14	15.2	13	14
TH01	5.3	8.3	8.3		8.3		5.3	8.3
D13S317	8	12	8	13	8	13	8	
D16S539	12	12.2	11		11	12	11	12.2
D2S1338	20		20	25	20	25	20	25
D19S433	12	14	11	11.2	11	12	11.2	14
VWA	15	15.2	13	18	13	15.2	13	15.2
TPOX	8	9	8	11	9	11	8	
D18S51	12	14	12	14	12	14	14	
AMEL	x	y	x		x		x	
D5S818	9	12	9	11	9		9	
FGA	23	24	24	25	24	25	23	25

**Table 4:** Quantification of wild-type and mutant *CYP21A2* clones

	Mother		S-1 (patient)		S-2	
	c.1069C >T, p.R357W (Exon 8)	c.1360C >T, p.P454S (Exon 10)	c.1069C >T, p.R357W (Exon 8)	c.1360C >T, p.P454S (Exon 10)	c.1069C >T, p.R357W (Exon 8)	c.1360C >T, p.P454S (Exon 10)
Total clones	94	117	118	55	58	83
Wt clones	5	8	3	0	2	0
Mut clones	89	109	115	55	56	83
% of Wt clones	5.3%	6.8%	2.5%	0	3.4%	0

Molecular characterization of *AQP2* mutation. Since *AVPR2* mutation is frequently found in male patients in X-linked recessive NDI and our patient is female, we screened for *AQP2* mutation first by analyzing all the 4 coding exons and intron-exon boundaries of AQP2 (NM_000486). PCR primers and conditions were described previously [11]. The resulting PCR products were directly sequenced using an automated ABI PRISM 3700 sequencer. A homozygous c.439G >A, p.A147T in exon 2 of *AQP2* was found in the patient (Fig. 1b). Her parents were heterozygous carriers and had no clinical symptoms or biochemical abnormality of NDI, indicating autosomal-recessive transmission. No genotype-phenotype discordance was observed for NDI. The mutation has been described previously to cause significant increase in water permeability [16].

## Discussion and conclusions

In the present study, we have characterized a family with genotype-phenotype discordance in 21OHD with concomitant NDI in the index patient. Genetic mosaicism at the *CYP21A2* locus has been identified and may contribute to the phenotype heterogeneity of 21OHD.

Our patient presented as a simple virilizing form of CAH although she had an aldosterone deficiency (increased renin and decreased aldosterone level). Based on published CAH nomogram on the analysis of 17-OHP levels among C21OHD, NC21OHD, and carriers, the phenotype of our patient is consistent with NC21OHD [1]. Mutant genotypes of R357W/R357W (previously named R356W) and P454S/P454S (previously named P453S) were found in the patient. Homozygous p.R357W is mainly associated with salt-wasting form of 21OHD and results in complete loss of its enzymatic activity [8, 17]. P454S is associated with NC21OHD [18]. The apparent genotype-phenotype discordance cannot be explained by the residual enzymatic activity or presence of rare unreported mutations since we have sequenced all the coding exons and intron-exon boundaries of *CYP21A2*. It is likely that small amount of wild-type *CYP21A2* alleles (2.5%) present in our patient cause phenotype variation and prevent development of salt-wasting form of 21OHD. The normal or milder phenotype seen in the mother and S-2 is probably due to higher percentage of wild-type *CYP21A2* alleles in their genome. It is unusual for the patient to carry two homozygous mutant genotypes (R356W and P454S), since majority of CAH patients are compound heterozygotes and NC21OHD phenotype is typically observed in these individuals. This may reflect high degree of consanguinity in the Turkish population.

It has been reported that genotype-phenotype discordance may be due to presence of previously unreported mutations [19] or certain mutations can cause different CAH phenotypes such as P30L, I172N, and I2G (g.655A/C > G) [8]. In the present study, we have identified genetic mosaicism as an alternative mechanism for genotype-phenotype discordance. Genetic mosaicism refers to the occurrence of two or more populations of cells with different genotypes within an individual. It can be either germline mosaicism developed from a single fertilized egg or somatic mosaicism derived from a postzygotic mutation. Genetic mosaicism should be considered in patients with poor genotype-phenotype correlation. Theoretically, mosaicism should not be transmitted from mother to child because mature eggs are in “all or none” situation (either of wild-type or mutant). Here we demonstrate mosaicism in both mother’s and her daughters’ (S-1 and S-2) somatic cells. One possible scenario is that the mosaicism found at c.1069 C >T (p.R357W) of *CYP21A2* locus is probably due to partial somatic gene conversion of 21-hydroxylase pseudogene *CYP21A1P* to *CYP21A2* during postzygotic mitosis. As shown in Fig. 1a, the corresponding *CYP21A1P* pseudogene sequence for c.1069C>T (p.R357W) is heterozygous c.1062C and c.1062T alleles present in the mother, S-1 and S-2; and homozygous c.1062C allele in S-3 (father is a homozygous carrier of c.1062T), which may explain the detection of small amount of wild-type c.1069C allele in the mother, S-1 and S-2: partial gene conversion of pseudogene c.1062C allele to wild-type c.1069C allele of *CYP21A2*. Interestingly, the corresponding *CYP21A1P* pseudogene sequence for c.1360C>T (p.P454S) is homozygous c.1353C in all the family members. Although the mother is mosaic for c.1360C locus containing both wild-type c.1360C and mutant c.1360T alleles, only mutant c.1360T allele is detected in S-1 and S-2, and wild-type c.1360C allele in S-3 (Table 4), which is consistent with the notion that mosaicism could not be transmitted from mother to child.

It is known that sensitivity of Sanger sequencing to detect minor allele or mutant allele is about 20–30% [20]. To reliably detect minor allele, its frequency should be at least 20%, which explains why the low frequency of wild-type alleles could not be detected by direct PCR-sequencing analysis. The PCR-based sequencing of individual clones or whole-exome sequencing may uncover many more cases of genetic mosaicism.

The patient also developed hyponatremia with polyuria and polydipsia at 13 months old during steroid replacement therapy, probably caused by *AQP2* mutation. The *AQP2* encodes a water channel protein located in the kidney collecting duct principal cells and plays an important role in water homeostasis. The activity of aquaporin 2 (AQP2) is regulated by the antidiuretic hormone vasopressin that determines water permeability in the kidney collecting ducts [21]. Disruption of this physiological mechanism by mutations in either *AVPR2* or *AQP2*, results in congenital nephrogenic diabetes insipidus (NDI) [22]. More than 278 different *AVPR2* and 64 *AQP2* mutations have been reported in patients with NDI (The Human Gene Mutation Database, HGMD) [23, 24]. The A147T in *AQP2* found in our patient has been reported to cause very low water permeability [25]. Concurrent *AQP2* and *CYP21A2* mutations have not been reported in the literature. Given that the patient carry three homozygous mutations: R357W and P454S in *CYP21A2* and A147T in *AQP2*, it indicates high degree of consanguinity in the patient family or Turkish population in general.

In conclusion, we have characterized a family with genotype-phenotype discordance of 21OHD with concomitant nephrogenic diabetes insipidus in the index patient. Genetic mosaicism may contribute to the phenotype heterogeneity of 21OHD. The concurrent *AQP2* and *CYP21A2* mutations result in CAH and NDI, respectively.

## Abbreviations

17-OHP: 17-hydroxyprogesterone
21OHD: 21-hydroxylase deficiency
ACTH: Adrenocorticotropic hormone
C21OHD: classic form of 21OHD
CAH: Congenital adrenal hyperplasia
NC21OHD: non-classic form of 21OHD
NDI: Hereditary nephrogenic diabetes insipidus
